# Prophylactic Uterine Artery Embolization Assisted Cesarean Section for the Prevention of Intrapartum Hemorrhage in High-Risk Patients

**DOI:** 10.1007/s00270-014-0855-8

**Published:** 2014-02-13

**Authors:** Qun Li, Zheng-Qiang Yang, Wasif Mohammed, Yao-Liang Feng, Hai-Bin Shi, Xin Zhou

**Affiliations:** 1 Department of Interventional Radiology, First Affiliate Hospital of Nanjing Medical University, 300 Guang Zhou Road, Nanjing, 210029 Jiangsu Province China; 2 Department of Obstetrics, First Affiliate Hospital of Nanjing Medical University, 300 Guang Zhou Road, Nanjing, 210029 Jiangsu Province China

**Keywords:** Uterine artery embolization, Intrapartum hemorrhage, Abnormal placentation, Hysterectomy, Cesarean section

## Abstract

**Purpose:**

To evaluate the safety and efficacy of prophylactic uterine artery embolization (UAE)-assisted cesarean section for the prevention of intrapartum hemorrhage.

**Materials and Methods:**

Twelve consecutive pregnant women (mean age 31 years; range 25–38) with uterine scarring and placenta previa and/or placenta accreta underwent UAE in conjunction with cesarean section to prevent intrapartum hemorrhage. For UAE, the left uterine artery was catheterized prophylactically under fluoroscopic guidance before the cesarean section incision was made. After the infant had been delivered, bilateral UAE was performed with the placenta still in situ. After successful bilateral UAE, the placenta was detached from the uterine wall.

**Results:**

Technical success was achieved in all 12 cases. Ten patients retained their uterus, and the other 2 underwent hysterectomy. The mean operative blood loss was 1,391 mL (range 600–3,600 mL). The total mean fluoroscopy time and mean absorbed dose (air kerma) were 9 min 40 s (range 4 min 35 s–15 min 24 s) and 91.79 mGy (range 30.2–171), respectively. The average fetal fluoroscopy time was 1 min 42 s (range 41 s to 3 min 16 s) with an average X-ray dose of 17.66 mGy (range 6.04–23.90).

**Conclusion:**

UAE-assisted cesarean section is safe and effective in the prevention of intrapartum hemorrhage in patients with uterine scarring and/or placental abnormalities.

## Introduction

Abnormal placentation, which broadly comprises placenta previa, accreta, increta, and percreta, is a leading cause of massive peripartum hemorrhage [1]. It frequently leads to emergency hysterectomies, surgical manipulation of organs adjacent to the uterus (ureter and bladder), respiratory distress syndrome, kidney failure, and even death [2, 3]. Blood loss in cesarean sections among patients with placental accreta can vary from 3,000 to 5,000 mL [4]. These conditions have been associated with significant maternal morbidity and mortality, and their occurrence has been reported to be increasing in recent decades [1, 5, 6].

Recently prophylactic hypogastric artery occlusion has been introduced to decrease intraoperative blood loss in patients at high risk for peripartum hemorrhage [4, 7]. The procedure consists of bilateral placement of embolizing materials and/or balloon catheters by way of the femoral arteries under fluoroscopic guidance. The literature offers evidence on the utility of preoperative placement of hypogastric artery catheters in decreasing blood loss and improving surgical outcomes [4, 7]. However, radiation exposure to the fetus and uterus during preoperative placement of balloon catheters or angiocatheters remains controversial. In addition, detailed data on fetal radiation exposure and maternal radiation exposure to the uterus have not been reported. Furthermore, transfer of the patient from the angiography suite to an operating room after prophylactic catheter placement will increase the complexity and risk of the procedure, particularly in unstable patients [8].

In this study, we investigated the safety and efficacy of uterine artery embolization (UAE) with preoperative catheterization in the unilateral uterine artery for assisted cesarean section for preventing intrapartum hemorrhage in patients with uterine scarring and/or placental abnormalities. The fetal and maternal radiation exposure doses were also recorded and analyzed.

## Materials and Methods

Our Institutional Review Board approved this study. Between February 2012 and 2013, the patient cohort consisted of 12 parturients ranging in age from 25 to 38 years (mean 31) who had placental accreta and/or placenta previa on ultrasonic Doppler and/or magnetic resonance imaging during the prenatal period. All patients had a history of at least one previous cesarean section. Their average number of previous pregnancies was 3.6 (range 2 to 8). All individuals had a clinical diagnosis of a potential high risk for massive intrapartum hemorrhage because of the abnormal placenta; the average gestational duration was 36 weeks (range 31 to 39; Table 1).

**Table 1:** Summary of patient characteristics

Patient no./age(y)	GA (week + day)	No. of previous curettages	No. of previous cesarean sections	MRI or US findings
1/30	36	4	1	Placenta accreta and placenta previa
2/38	35+5	8	1	Placenta percreta and placenta previa
3/35	35+2	3	1	Placenta previa
4/31	33+2	3	1	Placenta accreta and placenta previa
5/33	35+1	3	1	Placenta previa
6/31	35+2	2	1	Placenta accreta and placenta previa
7/35	33	8	1	Placenta accreta and placenta previa
8/33	34+1	3	1	Placenta previa
9/26	35+5	4	1	Placenta accreta and placenta previa
10/30	35	7	1	Placenta accreta and placenta previa
11/27	39+5	2	1	Placenta previa
12/27	31+2	3	1	Placenta accreta and placenta previa
Mean/31.3	35+2	4	1	–

*GA* gestational age

**Fig. 1:**
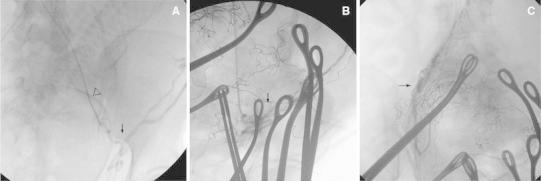
Fluoroscopy images during procedure: Steps of prophylactic UAE-assisted cesarean section procedure in a 26-year-old patient (case no. 9). **A** A uterine catheter was inserted into the left uterine artery (*arrow*) superselectively before delivery. The fetus (*arrowhead*) is located within the uterus. **B** The left uterine artery (*arrow*) can be seen after successful embolization with ample gelfoam and contrast medium deposition. In addition, note the multiple shadows of hemostats used to stop bleeding after delivery. The placental outline can also be seen because it was still attached to the uterine wall. **C** The right uterine artery (*arrow*) can be seen after selective catheterization and embolization after infant delivery and before placental separation

Before surgery, all patients provided written informed consent and were brought to a regular operating room fitted with a moveable C-arm equipped with digital subtraction angiography (DSA; Philips Medical Systems, Best, Netherlands) and a conventional operating table. After anesthetizing the patient with an epidural block, preoperative placement of temporary bilateral ureteral stents under cystoscopy and vesical catheter insertion were performed. In each patient, a 5F sheath was placed in the right common femoral artery using Seldinger technique. Selective catheterization of the left uterine artery was performed with a 5F Roberts uterine curve (RUC) catheter (Cook, Bloomington, IN) under fluoroscopic guidance. The cumulative radiation dose and fluoroscopy time during the selective catheterization of the left uterine artery were recorded with the fetus in situ. The cumulative radiation dose absorbed per patient (air kerma) was recorded using the DSA system during fluoroscopy. Then a traditional cesarean delivery procedure was performed. After the infant had been delivered and the umbilical cord clamped, the obstetrician clipped any bleeding vessels using hemostatic forceps and packed the vagina and the uterus with the placenta still in situ (Fig. 1).

Under fluoroscopic guidance, the left uterine artery was embolized first, following which the right uterine artery was selectively catheterized and embolized with Gelform sponge pledgets (absorbable gelform sponge; Jinling pharmaceutical company, Nanjing, China) mixed with contrast medium. Both uterine arteries were embolized until there was adequate stasis, and the catheter and sheath were kept stationary in case further embolization was necessary. The cumulative radiation dose and fluoroscopy time during bilateral UAE were recorded, and the complete placenta was removed manually from the uterine wall using the traditional caesarean section technique. If bleeding continued even after bilateral UAE and led to an unstable blood pressure, emergency hysterectomy was performed.

After closing the uterine lumen and abdominal cavity and observing no vaginal bleeding, the angiocatheter was removed under fluoroscopic guidance. The puncture point of the right femoral artery was compressed for more than 10 min for hemostasis. Intraoperative blood loss and volumes administered to the patient (crystalloid, colloid, and blood) were recorded. The length of surgery, infants’ Apgar scores, and any complications after the procedure were recorded.

## Results

No fetal or maternal mortality occurred in this group. Two parturients had refractory postpartum hemorrhage that was controlled by bilateral UAE followed by hysterectomy. The first of these patients had a large placenta previa that even covered the cervical os, and the second patient had placenta percreta, which extended into the uterine serosa and the wall of bladder. In 11 of 12 cases, the infant’s Apgar score was 8 to 10 at 1 and 5 min, respectively; the remaining neonate scored 5 at birth, but the score increased to 8 at 5 min after cardiopulmonary resuscitation. In 7 of 12 cases, the average fluoroscopy time used to place the RUC catheter was 1 min 42 s (range 41 s to 3 min 16 s). The average X-ray dose was 17.66 mGy (range 6.04–23.90), and the average dose area product was 0.493 mGym^2^ (range 0.174–0.709). For the first five cases, we only recorded total fluoroscopy time and dose. The average total duration of fluoroscopy was 9 min 40 s (range 4 min 35 s to 15 min 24 s). The average dose was 91.79 mGy (range 30.2–71), and the average dose area product was 2.37 mGym^2^ (range 0.849–4.09).

The average blood loss during the surgical procedure of all 12 cases was 1,391 mL (range 600–3,600), and the average urinary amount was 358 mL (range 200–500). The average volume of liquids administered was 2,333 mL (range 800–7,000). The average volume of colloid administered was 393 mL (range 200 to 910), and the average amount of blood transfused was 4.875 units (range 2 to 13) of packed red blood cells. The average duration of the surgical procedure was 2 h 19 min (range 1 h 20 min to 3 h 30 min; Table 2). No further complications, such as infection and hemorrhage, were found in any of the cases. All patients had normal pulsation in the dorsal artery of the foot. The volume of vaginal bleeding was approximately 80 mL (range 20–150). The mean postoperative hospitalization time was 8.75 days (range 7–10). The documentation for every patient was complete.

**Table 2:** Summary and outcomes of surgical and radiological procedures

Patient no.	Hysterectomy	Fetal fluoroscopy time (min:s)	Fetal fluoroscopy dose (mGy)	Fetal dose area product (mGym^2^)	Total fluoroscopy time (min:s)	Total fluoroscopy dose (mGy)	Total dose area product (mGym^2^)	Estimated blood loss (ml)	Replenishment total fluid volume (ml)	Replenishment colloids fluid volume (ml)	Transfused packed red blood cells (units)	Surgery Time (hours :mins)	Apgar score
1	N	N/A	N/A	N/A	4:35	30.2	0.85	1,600	1,500	370	4	01:30	10
2	Y	N/A	N/A	N/A	5:58	61.2	1.74	3,600	7,000	910	13	03:30	10
3	N	N/A	N/A	N/A	9:13	77.5	2.00	1,500	1,500	270	6	02:10	10
4	N	N/A	N/A	N/A	15:24	171	4.09	1,000	2,500	530	5	03:30	10
5	N	N/A	N/A	N/A	13:12	100	2.90	800	1,500	540	2	01:30	10
6	N	1:13	18.5	0.428	14:45	156	3.12	800	1,500	400	–	02:10	Twins 9–10
7	Y	1:38	18	0.534	10:34	109	3.21	2,500	5,000	700	10	02:40	9–10
8	N	3:16	23.9	0.709	10:20	87.7	2.59	2,000	1,500	800	7	03:00	5 at 1 min 8 at 5 min
9	N	1:24	16.7	0.494	9:28	100	2.94	600	2,000	–	2	01:50	10
10	N	1:53	18.8	0.552	5:36	56.9	1.41	800	1,500	–	3	01:20	10
11	N	1:51	21.7	0.561	5:05	56	1.36	900	1,500	–	4	02:40	10
12	N	0:41	6.04	0.174	11:52	96	2.27	600	1,000	200	2.5	02:00	9
Mean	16.7 %	1:42	17.66	0.493	9:40	91.79	2.37	1,391	2,333	393	4.875	02:19	10

*N/A* data not available, *Y* yes, *N* no

## Discussion

In this study, we showed that UAE-assisted cesarean section is an effective and safe method that is useful in the prevention of massive intrapartum hemorrhage in patients with abnormal placenta. In 10 of 12 patients, the uterus were successfully preserved with complete placental removal after prophylactic bilateral UAE.

Previous studies on conventional hysterectomy in similar patients found massive blood loss [9, 10]. Eller et al. identified 76 cases of patients with placenta accreta who underwent hysterectomy and reported that mean blood loss was ~2.6 L [11]. Compared with those reports, there was much less blood loss (average 1,391 mL) in our study.

Weeks et al. [4] reviewed 21 patients undergoing hysterectomy who also underwent perioperative temporary balloon iliac artery occlusion. Although balloon occlusion was performed before hysterectomy, there was still an estimated blood loss of 1,671.5 mL. Another report employing similar methods reported estimated blood losses in successful cases ranging from 500 to 2,300 mL [8], which is similar to our study. However, in the same study, there were 6 cases (54 %) with the placenta left in situ, which might have led to a high incidence of recidivism. There was also massive blood loss in the two failed cases (13,000 and 10,000 mL). The timing of insertion of the RUC catheter—after delivery of the baby in that study [8] compared with before delivery in our study—is the primary difference between the two reports. Delayed RUC insertion after delivery of the baby can lead to a delay in embolization, which could explain the higher volume of postpartum hemorrhage.

We found that fetal radiation exposure was minimal because only a single unilateral uterine artery was catheterized selectively under fluoroscopic guidance while the fetus was in utero. According to the National Council on Radiation Protection and Measurements, the risk to an infant from fluoroscopy dosage is increased significantly when the radiation exposure exceeds 150 mGy [12, 13]. However, at doses of <50 mGy, the risk of radiation-induced abnormality is considered negligible compared with baseline risks for all developmental abnormalities, e.g., 1 % for mental retardation and 3 % for spontaneous birth defects [12, 14]. There was a wide range of total radiation dose and time because in some cases secondary UAE or other pelvic artery embolizations were performed when the obstetrician found further intraoperative hemorrhage during placental separation. It was convenient and flexible for us to perform repeat embolization because the DSA machine was stationed in the regular operating suite and the catheterization had been preserved.

Major complications associated with UAE in obstetric patients occur rarely with an overall complication rate of ~9 % [8]. These include transient fever, pelvic pain, transient foot ischemia, transient menelipsis, and abscess. In our study, all mothers had normal pulsation of the dorsal artery of the foot, and the average volume of vaginal bleeding was only ~80 mL. The mean hospitalization time was 8.75 days. Compared with a perioperative temporary occlusion study, which reported thrombogenesis in the femoral artery and other complications [4], our method seems to be much safer.

Another important innovation of our study was the timing of RUC insertion into the uterine artery. Some previous investigators did not use fluoroscopy before fetal delivery. Instead, they inserted a 5F femoral arterial sheath [8, 15] before delivery to minimize fetal radiation. However, the main causes of UAE failure in these studies were vascular spasm with shock resulting from massive blood loss along with uterine artery movements during delivery, which made it difficult to accurately place the RUC into the uterine artery. Although our method of placing the RUC before delivery is simple and safe, we still must consider the decreased blood flow associated with fetal distress and the increased fluoroscopy exposure time if the RUC is placed in both uterine arteries. Hence, we elected to catheterize one unilateral uterine artery only before delivery to decrease the exposure time, and the Apgar score of all but one of the delivered fetuses was within the normal range.

A major limitation of our research was that it was retrospective in nature and the fetal fluoroscopy dose was only available for seven cases. Furthermore, this method must be validated by prospective controlled studies before mainstream implementation. Although we showed that there was minimal radiation exposure to the fetus during the positioning of the uterine artery RUC, which is below the specific threshold radiation level for teratogenesis, carcinogenesis is a stochastic effect of radiation, and there is no threshold level below which the risk of cancer can be eliminated completely [8, 12]. Hence, the safety of this method remains to be established by long-term studies.

In recent years, there has been significant interest in the potential role of interventional techniques in obstetrics, specifically in the control of peripartum hemorrhage [16]. Great attention has been paid to the potential of these procedures in improving maternal outcomes by decreasing morbidity and mortality. In our study, the blood loss volume was effectively controlled in most cases with an acceptable fluoroscopy dose. However, future randomized controlled trials and long-time follow-up studies are needed to determine whether the potential benefits of UAE during delivery outweigh the potential risks associated with fetal radiation exposure and vascular complications.
